# Evolution of Sex Determination Loci in Atlantic Salmon

**DOI:** 10.1038/s41598-018-23984-1

**Published:** 2018-04-04

**Authors:** James Kijas, Sean McWilliam, Marina Naval Sanchez, Peter Kube, Harry King, Bradley Evans, Torfinn Nome, Sigbjørn Lien, Klara Verbyla

**Affiliations:** 1 CSIRO Agriculture & Food, St. Lucia, 4067 QLD Australia; 2 CSIRO Agriculture & Food, Hobart, 7004 TAS Australia; 3 Tassal Operations, Hobart, 7000 TAS Australia; 4 Centre for Integrative Genetics (CIGENE), Department of Animal and Aquacultural Sciences, Faculty of Biosciences, Norwegian University of Life Sciences, P.O. Box 5003, N-1432 Ås, Norway; 5 CSIRO Data 61, Canberra, 2601 ACT Australia

## Abstract

Teleost fish exhibit a remarkable diversity in the control of sex determination, offering the opportunity to identify novel differentiation mechanisms and their ecological consequences. Here, we perform GWAS using 4715 fish and 46,501 SNP to map sex determination to three separate genomic locations in Atlantic salmon (*Salmo salar*). To characterize each, whole genome sequencing was performed to 30-fold depth of coverage using 20 fish representing each of three identified sex lineages. SNP polymorphism reveals male fish carry a single copy of the male specific region, consistent with an XX/XY or male heterogametric sex system. Haplotype analysis revealed deep divergence between the putatively ancestral locus on chromosome 2, compared with loci on chromosomes 3 and 6. Haplotypes in fish carrying either the chromosome 3 or 6 loci were nearly indistinguishable, indicating a founding event that occurred following the speciation event that defined *Salmo salar* from other salmonids. These findings highlight the evolutionarily fluid state of sex determination systems in salmonids, and resolve to the sequence level differences in animals with divergent sex lineages.

## Introduction

The genetic and environment factors that contribute to sexual determination are of fundamental biological interest, both from an evolutionary perspective and within an aquacultural production context. Teleost fish are of particular interest in both domains. They have evolved a spectacularly diverse array of sex determination systems, with examples spanning strict genetic control, total environmental control and complex combinations that interpret both genetic and environmental cues to dictate sexual trajectories. A number of species have been developed into globally important production species, and where there are gender-related differences in commercial value, knowledge concerning sexual determination is required to manipulate sex ratios and enhance production efficiency.

Unlike mammals which share a master sex determining (SD) gene (*SRY*^1^), at least five genes have been identified that control SD in fish including *amhy*, *amhr2*, *dmY*, *gsdf* and *sdY*^2^. Each acts differently, however control over the initiation and rate of cell proliferation appears to be a common feature. The genetic control of sex determination in salmonids involves *sdY* (sexually dimorphic Y chromosome), and is thought to employ a male heterogametric (XY) system^3^. The mechanistic action of *sdY* is less clearly understood than for other fish master SD genes, as it has not previously been implicated with sexual development in other species. The gene is male specific in 13 different salmonid species which strongly suggests it has a controlling function^3,4^. Comparison of the genomic location of the SD locus between salmonids indicates it is often non-syntenic. This evoked the concept that *sdY* is mobile and through speciation has taken unique chromosomal positions^5,6^. Interestingly, investigation within Atlantic salmon mapped *SEX* to multiple chromosomes and the presence of *sdY* is very strongly (but not perfectly) correlated with maleness^7,8^. Multiple locations for *sdY* within Atlantic salmon suggests that the mechanism driving it’s genomic mobility have continued to occur following the speciation event founding Atlantic salmon. The alternative is that multiple genomic locations predated the radiation of Atlantic salmon and balancing evolutionary pressure has maintained multiple sex lineages to the present.

In this study we characterise the number, location and evolutionary history of sex determination loci in Atlantic salmon. Samples were chosen from a large pedigreed population of farmed Tasmanian animals where data describing the sex of each animal is routinely collected as part a breeding program. Tasmania is outside the natural range of the species, and the study population originates from North American wild stocks^9,10^. To commence their genetic characterisation, we performed whole genome sequencing of 20 animals and compared genome wide levels of nucleotide diversity and allele frequency correlation with other populations. This showed the study population retains comparable diversity to other farmed salmon, and have patterns of SNP polymorphisms consistent with a North American ancestry. To determine the number of sex determination loci present, we performed a genome wide association study using 4716 individuals genotyped with a 50 K SNP array. We confirmed the presence of three separate genomic regions controlling sex, and report association leakage that we propose is a residual consequence of the incomplete rediplodization of the salmon genome. To characterise the evolutionary history of each SD locus, genome sequence from animals with known sex lineage were used for read mapping and variant detection. This defined the boundary of the male specific region and confirmed males carry a single copy consistent with male heterogameity. Haplotype analysis suggests two events have shaped the evolutionary history of sex determination. We identify the likely ancestral sex determination locus, and two descendent lineages of which one has likely been founded after the speciation event that defines Atlantic salmon.

## Results

### Sequencing, Variant Detection, Nucleotide Diversity and Population Divergence

**Figure 1:**
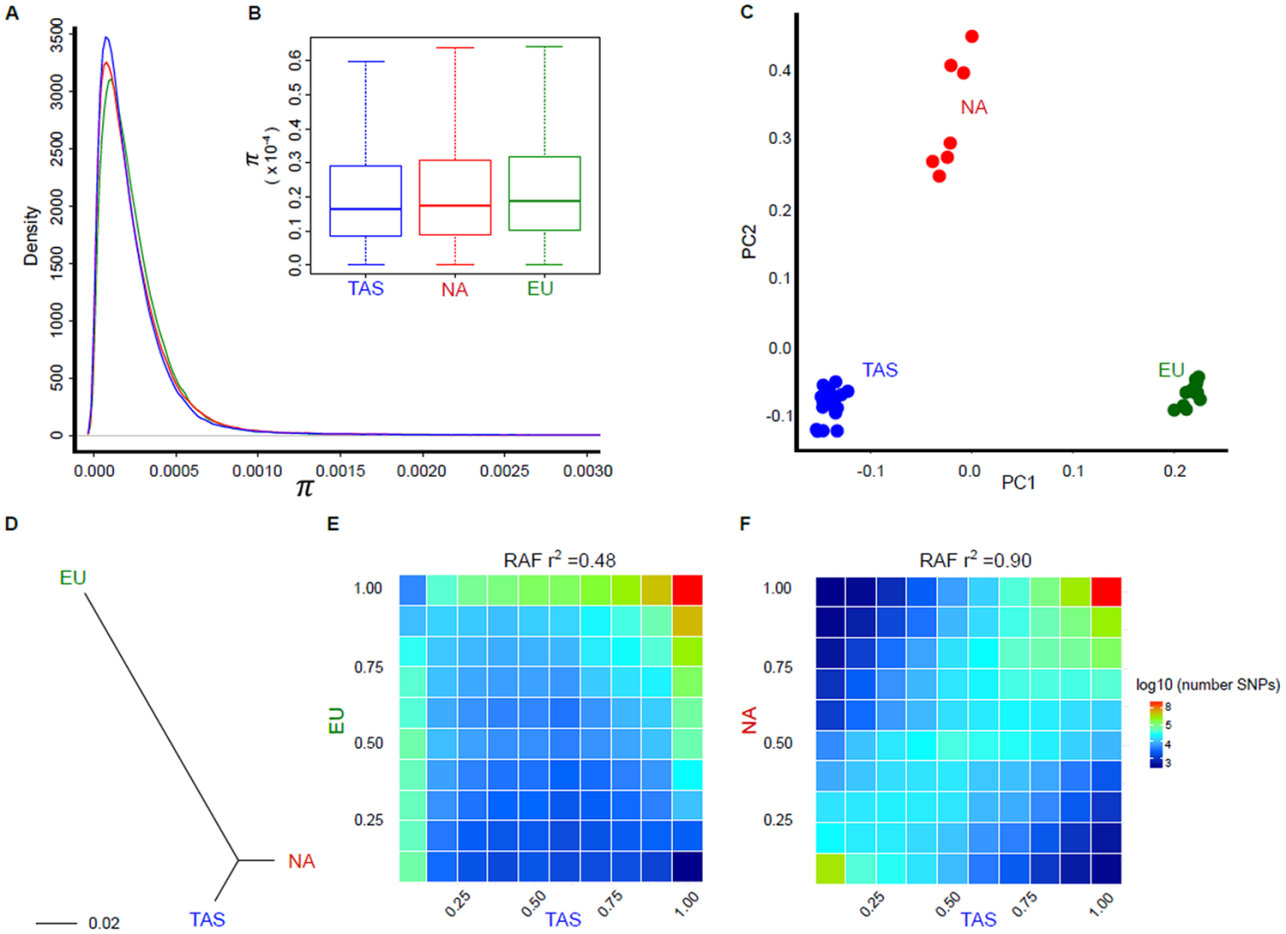
Population diversity and divergence. (**A**) Nucleotide diversity was estimated in 20 Kb genomic intervals for the TAS, EU and NA populations. (**B**) Following subsampling of the TAS data to match the read depth available for North American (NA) and European (EU) salmon genomes (S2 Fig), nucleotide diversity is shown as a box and whisker plot. (**C**–**F**). Three methods illustrate the divergence between populations. First, PCA of genetic distance (**C**) was performed to assess the clustering of animals, coloured according to their population. Next, pairwise population *F*_ST_ was used to construct a dendogram linking the populations (**D**). Finally, Reference allele frequency (RAF) bins were estimated within each population, before being compared between populations and visualised as heatmaps. SNP displaying low RAF in one population and high RAF in second are located at the top left and bottom right of the heatmap. Conversely, SNP with highly correlated RAF are located on the axis joining the bottom left and top right. Increasing SNP count is indicated using increasingly warm color. The correlation of RAF for the TAS and EU (**E**), and TAS and NA (**F**) is given above each heatmap.

To measure genome diversity and characterise sex determination loci in Tasmanian Atlantic salmon (TAS), we performed whole genome sequencing (WGS) of 20 fish selected from a range of year classes and families. Read mapping against reference genome ICSASG_v2^11^ resulted in 33–48 fold depth of coverage per animal (Table 1). Variant calling identified 10.11 M SNP, before quality filters were applied to remove one sample and identify a final collection of 8.92 M high quality SNP. Filtering included the identification and removal of 1.19 M positions unlikely to be SNP, due to their presence within the genomes of four female double-haploid fish (Supplementary Fig. S1). Accuracy of our WGS variant calling pipeline was assessed by comparison against array based genotypes from six animals. Comparison at 16,346 loci returned 99% concordance rate, confirming preparation of a high quality WGS SNP collection. Analysis of genome wide nucleotide diversity (π) measured in 20 Kb genomic bins revealed moderate levels of polymorphism (average π = 2.31 × 10^−4^, Fig. 1A). To assess the relative level of diversity in comparison to other farmed salmon populations, public WGS datasets were obtained from North American (NA) and European (EU) derived strains of Atlantic salmon cultured in Chile^12^. Given the depth of coverage available was approximately four times deeper in the TAS genomes, corrective read subsampling was performed before variant calling was repeated using matched lower depth of coverage (refer to the materials and methods). The effect of subsampling on the proportion of shared SNP between populations (TAS, EU and NA) is given in Supplementary Figure S2. The approach allowed a direct comparison of nucleotide diversity between populations (Fig. 1B), revealing the TAS population is slightly less diverse than other NA derived (average π = 2.43 × 10^−4^) and European derived farmed animals (average π = 2.44 × 10^−4^). All three population estimates are based on small sample sizes and are likely to change as more data become available, however the analysis revealed the TAS breeding population, closed since the importation of animals into Australia in the 1960s, has retained diversity comparable to other aquaculture strains. Relatedness between individual genomes was explored using Principal Component Analysis (PCA) of pairwise genetic distance (Fig. 1C). Individuals formed clearly distinct population clusters, and the largest principle component (PC1, 2.62% of variation) positioned each Tasmanian salmon (strongly negative PC1 values) closer to North American animals (slightly positive PC1 values) when compared with European derived fish (strongly positive PC1 values). PC2 (1.55% of variation) distinguished two groups of NA fish, corresponding to animals derived from either Nova Scotia or Quebec. The cluster from Nova Scotia are closer to the TAS animals, consistent with a shared geographic origin of their ancestors. In order to more accurately quantify the divergence separating populations, two additional metrics were estimated. First, the strength of allele frequency correlation was examined. The reference allele at each variant was defined as the nucleotide present within reference assembly ICSASG_v2. The number of SNP in reference allele frequency (RAF) bins were compared between populations and presented as a heatmap (Fig. 1). The TAS and NA populations contained highly correlated RAF (average correlation of *r*^2^ = 0.90, Fig. 1E). In contrast, the TAS and EU populations had lower correlation (*r*^2^ = 0.48) due to an increased prevalence of SNP with divergent RAF (Fig. 1F). Next, population divergence was estimated at each SNP using *F*_ST_, before the average value for each pairwise population comparison was used as input for construction of a dendogram (Fig. 1D). This revealed the lowest divergence was observed between the TAS and NA populations (*F*_ST_ = 0.041 ± 6.95 × 10^−5^). Divergence between European fish was very similar when compared with either the TAS (*F*_ST_ = 0.178 ± 1.04 × 10^−4^) or NA animals (*F*_ST_ = 0.172 ± 1.26 × 10^−4^). Taken together, the three analytical approaches (PCA, RAF and *F*_ST_) report findings entirely consistent with the known population history of the three populations.

**Table 1:** Salmon used for whole genome sequencing. Phenotypic assignment of sex (PSEX) is given along with the sex lineage (SL) assigned by either simple sequence repeat (SSR) segregation data^7^ or using whole genome sequence data in this study (SL_WGS). Missing data is indicated as ‘nd’. The depth of coverage and number of SNP per animals is given following application of quality filtering.

Animal	PSEX	SL_SSR	SL-WGS	Coverage	SNP
1_2012	M	Ssa06	Ssa03/06	44.0	6,927,595
2_2005	M	nd	Ssa03/06	48.5	7,010,428
3_2005	F	Ssa06	nd	42.0	6,901,270
5_2006	F	nd	nd	43.6	6,920,473
6_2007	M	Ssa03	Ssa03/06	42.8	6,926,011
7_2007	M	Ssa02	Ssa02	47.1	6,955,804
8_2007	F	Ssa03	nd	32.9	6,674,337
9_2009	F	Ssa06	nd	37.1	6,857,336
10_2013	M	Ssa06	Ssa03/06	28.9	6,567,065
11_2006	M	Ssa06	Ssa03/06	36.6	6,774,991
12_2007	M	Ssa06	Ssa03/06	36.2	6,743,704
13_2009	F	Ssa02	nd	33.6	6,762,881
14_2010	F	Ssa02	nd	38.0	6,796,035
15_2011	M	Ssa02	Ssa02	39.6	6,869,394
16_2012	F	Ssa06	nd	44.1	6,969,672
17_2013	M	nd	Ssa03/06	48.2	7,012,754
18_2013	M	Ssa03	Ssa03/06	44.7	6,959,577
19_2015	nd	nd	Ssa02	45.0	6,978,524
20_2015	nd	nd	nd	47.9	6,997,752

### Genome Wide Association Study for Sex Determination

**Figure 2:**
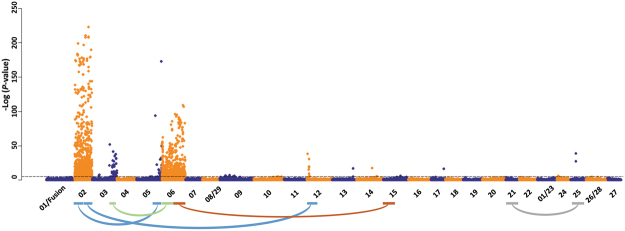
GWAS for sex determination. A total of 46,502 SNP were used for association analysis to identify sex determination loci in a population of 4715 fish scored for genotypic sex (GSEX). SNP associations are plotted in genomic order, with even numbered and odd numbered chromosomes shown in orange and blue respectively for a karyotype consisting of 27 autosomes. Homeologous regions for each of the 6 critical intervals (Table 2) are shown below the plot, with region pairs linked by curved lines. The same data is shown in expanded format or Ssa02 in Fig. S3.

To begin the characterisation of sex determination, GWAS was performed to i) determine the number of SD regions segregating within the TAS population and ii) map the location of each SD locus onto the reference assembly. To perform GWAS, a total of 4716 animals were genotyped using a custom SNP array. Animals were scored for phenotypic sex by visual assessment (PSEX, 3176 fish, 1772 female, 1404 male). Using the SNP array, animals were also assayed for the presence or absence of the *sd*Y gene to assign genotypic sex (GSEX, 4715 fish, 2856 female, 1859 male). Concordance between the two traits was high but not perfect (98%), and discordant individuals were excluded from further analysis. Following the application of genotype quality filters, a total of 46501 SNP were available for analysis. It is important to note array elements used in the assignment of GSEX were excluded. GWAS was performed using linear regression and a case control design before the strength of association to GSEX (Fig. 2) was plotted for SNP in genomic order. Strong association peaks were observed on six chromosomes (Ssa02, 03, 05, 06, 12, 25) while single isolated SNP were identified on four additional chromosomes (Ssa13, 14, 15 and 17). Three of the chromosomes with association peaks (Ssa02, 03 and 06) were reported previously in the Tasmanian population and earlier findings in European Atlantic salmon^7,13^. A chromosome specific threshold method was used to define the size and location of critical intervals for each association peak. Despite using more than 4000 animals with both SNP and categorical trait data, the size of each critical interval was persistently large. For example, the massive association signal on Ssa02 contained 884 loci spanning 33 Mb (or 45% of the chromosome) and contained the most strongly associated SNP (*AX-87161956*, p-value = 223, Mb position 50.74). Male recombination is strongly localized to some telomeric regions in salmonids^14^, which has likely contributed to our inability to accurately map sex determination. The size, location and peak position for each of the critical intervals is given in Table 2 and plotted separately in Supplementary Figure S3.

**Table 2:** Genomic intervals associated with sex determination. A chromosomal specific threshold (p-value) identifying the top 0.5% of associated SNP was used to define critical intervals (refer to methods). The size of each is given in Mb, along with the number of significant SNP and Mb position the interval starts and stops.

CHR	Size (Mb)	Start (Mb)	Stop (MB)	Peak (Mb)	Threshold	SNP
Ssa02	33.0	17.8	50.8	50.7	197	7
Ssa03	27.6	62.3	89.9	62.3	23	9
Ssa05	26.4	52.7	79.1	75.9	14	10
Ssa06	18.8	51.0	69.7	66.8	86	9
Ssa12	13.3	4.3	17.6	4.3	5	7
Ssa24	1.6	2.5	4.2	4.2	5	3
Ssa25	0.02	17.8	17.8	17.8	5	2

### Genome Duplication, Homeologs and Association Signal Leakage

The evolutionary history of Atlantic salmon is characterised by a salmonid specific whole genome duplication event. Approximately 25% of the genome has delayed rediploidization and chromosome region pairs retain sequence similarity >90%. The chromosomal location of these duplicated homeologous blocks, each comprising two non-syntenic regions, have recently been defined within the reference genome assembly^11^. Further, male recombination at telomeres with high sequence homology between homeologs is highly repressed, likely as a result of multivalent pairing at meiosis obstructing recombination^14^. To assess if the genome duplication and associated complexity might partly explain the identification of multiple SD loci, the homeologous partner region for each of the six GWAS critical intervals was identified (Fig. 2). The large critical interval on Ssa02 spans the boundary between homeologous blocks such that the 2p end of the interval (Mb position 18–30) is homeologous to Ssa05 (Mb 50–80) while the 2q end (Mb 30–50) is homeologous to Ssa12 (Mb 0–30). Both of these partner regions, on Ssa05 and 12, exactly match GWAS association signals for sex (Fig. 2, Table 2). Conversely, the Ssa03 SD critical interval (Mb 60–90) is homeologous to a region not implicated by the GWAS (Ssa6 Mb 0–40). The Ssa06 region is the same, as it’s partner region (Ssa15 Mb 15–55) was not associated with SD by GWAS.

**Figure 3:**
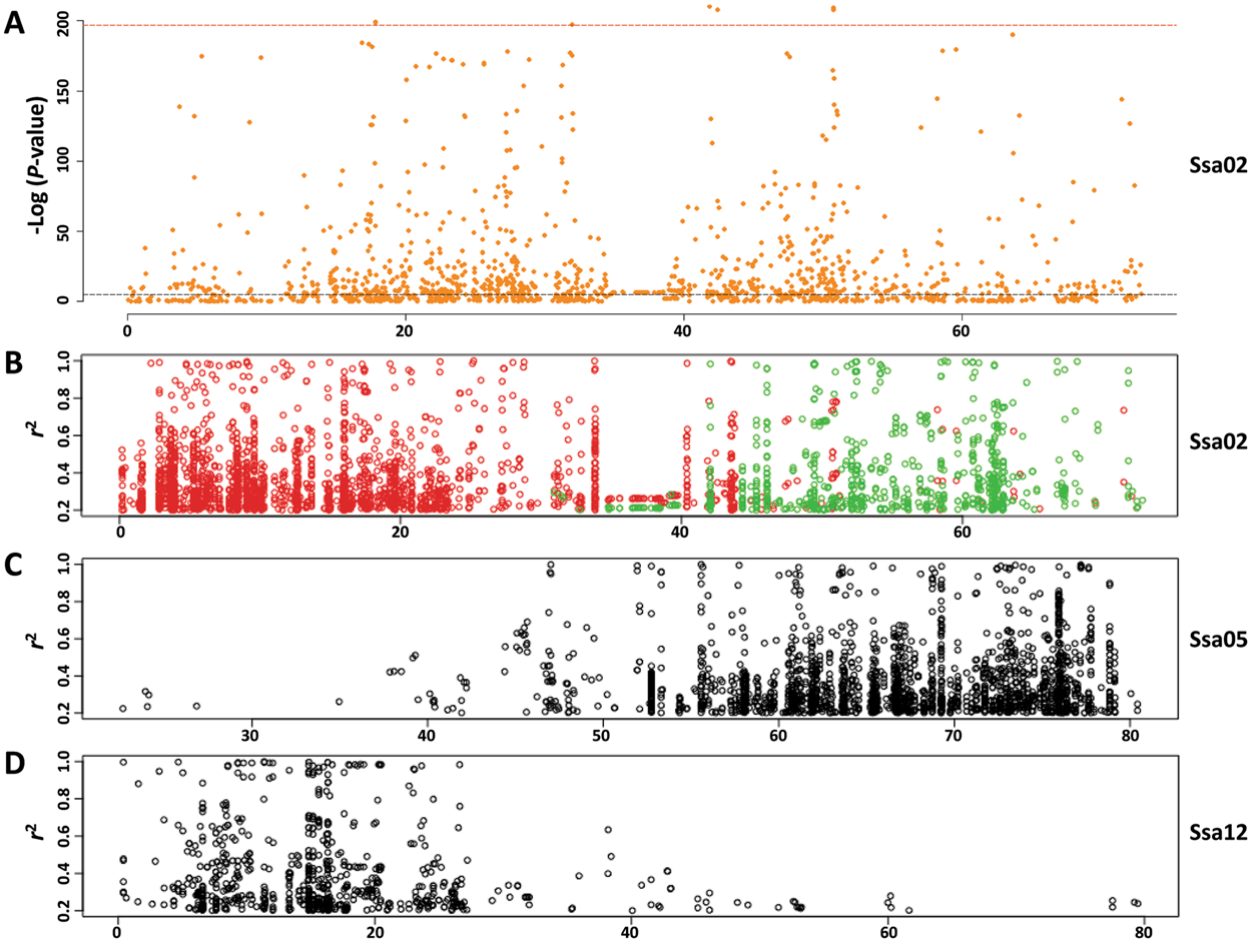
Non-syntenic linkage disequilibrium. Non-syntenic linkage disequilibrium corresponds with the genomic location of homeologous chromosome segments. (**A**) SNP associations to GSEX spanning Ssa02 are shown, along with the chromosome specific threshold defining the top 0.5% of SNP ranked on *P*-value. Marker locations are given as Mb and the data is a subset of that displayed in Fig. 2. Highly associated loci are located throughout, with the exception of the centromeric region spanning Mb 34–38. (**B**–**D**) Linkage disequilibrium for SNP pairs located on different chromosomes (non-syntenic). (**B**) SNP positioned on Ssa02 are shown that display elevated LD (*r*^2^ > 0.2) with loci on either Ssa05 (red) or Ssa12 (green). Note that only SNP with *r*^2^ > 0.2 are shown and the scale does not extend to zero. The location of SNP pairs with elevated non-syntenic LD aligns with the known homeologous chromosomal segments, as graphically depicted in Fig. 2. For example, the 2p end of Ssa02 (Mb 0–30) displays high LD with Ssa05 (Mb 50–80) while 2q (Mb 50–80) displays extensive LD with loci spanning Ssa12 (Mb 0–30). (**c**) SNP positioned on Ssa05 are shown with *r*^2^ > 0.2 with loci on Ssa02. (**D**) SNP positioned on Ssa12 are shown with *r*^2^ > 0.2 with loci on Ssa02.

The finding that association signals on Ssa05 and 12 both reside in homeologs of Ssa02 prompted deeper analysis to explore if they might be artefactual. If the GWAS signals on chromosomes 5 and 12 reflect true sex determination loci, the SNP generating the associations should assort independently to markers elsewhere in the genome. Independent behaviour was assessed using linkage disequilibrium (LD), which when measured as *r*^2^ should take values approaching zero for SNP pairs located on difference chromosomes^15^. Each SNP on Ssa02 had *r*^2^ estimated in pairwise combination with SNP on both Ssa05 and Ssa12, before values were plotted in chromosomal order (Fig. 3). This revealed 191 SNP situated throughout the telomeric half of Ssa02 (Mb 0–38) had extremely strong LD (*r*^2^ > 0.5) with 165 loci on Ssa05. Even higher non-syntenic pairs were observed for LD with *r*^2^ > 0.2 (520 and 482 loci on for Ssa02 and Ssa05 respectively). Similarly, 77 SNP distributed across the acrocentric half of the chromosome (Mb 40–80) had elevated LD with 90 loci on Ssa12 (247 and 315 loci on Ssa2 and Ssa12 at *r*^2^ > 0.2). This clearly demonstrated these homeologous regions do not behave independently, but rather have highly correlated allele frequencies despite being on different chromosomes. Importantly, each region has been implicated previously as likely to retain residual tetrasomy, potentially explaining the persistence of high LD^11^. No elevated LD was detected (*r*^2^ > 0.2) for SNP pairs residing on Ssa05 and Ssa12 (Fig. 3). Given these are not homeologs, it appears the elevated LD is confined to homeologous partner regions. These results raise the possibility that correlated non-syntenic allele frequencies are responsible for generating false positive association signals. In this case, the exceptionally strong true association signal detected on Ssa02 appears to have leaked to Ssa05 and Ssa12. The suggestion that the Ssa05 and Ssa12 signals are spurious is supported by the observation that neither chromosome was implicated by previous microsatellite segregation analysis of the Tasmanian population^7^ or by FISH experimentation using *sd*Y positive clones^9^. This type of association signal leakage has not been reported previously for other salmon GWAS where strong association was reported^16,17^. Nor was it detected during selection sweep analysis in a study that explicitly explored the potential consequences imposed by the whole genome duplication^18^. This can be explained where such signals reside outside the approximately 25% of the genome that appears to have experienced delayed rediploidization. None the less, the correlated allele frequencies reported here have the potential to generate false positive signals in GWAS.

### Identification of the male specific region and sex lineages from WGS

**Figure 4:**
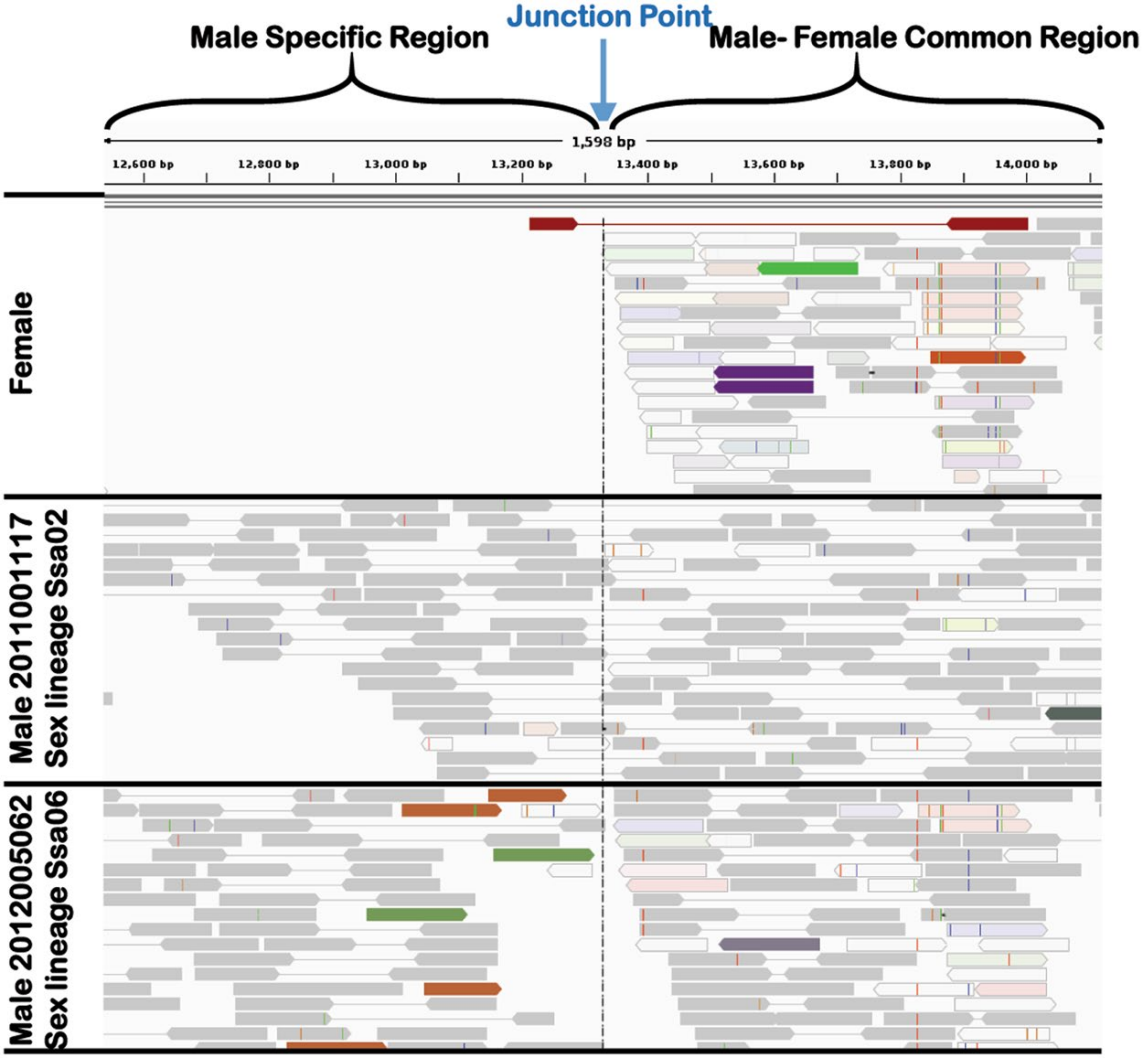
Sequence architecture surrounding the male specific region in three fish. Sequence reads from three fish identifying the junction point boundary of the male specific region. Reads positioned with high map quality in the correct orientation and spacing are identified as linked grey pairs. Reads mapping with high quality, but who’s mate is mapped elsewhere on the genome, are visualised as an unlinked read. The colour of unlinked reads identify the chromosomal location of their mate pair, with green signifying Ssa03 and brown Ssa06. Additional nomenclature and explanation is given by the developers of the Integrated Genome Viewer^26^. Read mapping is shown for three animals. A female (top panel) and two males.

Whole genome sequence data from 19 fish (Table 1) was used to characterise two aspects of Atlantic salmon’s sex determination loci; (i) the location and sequence context of the junction point separating the male specific region (MSR) from the male – female common region (MFCR) and (ii) if sequence data alone could distinguish between males belonging to different sex lineages. Given the reference genome assembly was derived from a double-haploid female, it is not expected to carry either the MSR or *sd*Y gene^11^. This prompted two groups to build and publish contigs spanning *sd*Y by sequencing three BAC clones isolated from a single male Norwegian fish^9,19^. A 20 Kb contig spanning *sd*Y^9^ was appended to reference ICSASG_v2, before raw read mapping was performed for each fish (see Materials and Methods). Read mapping and depth, when compared between male and female fish, precisely defined the boundary between the MSR and MFCR (Fig. 4). It is located at position 13,333, which is consistent with earlier PCR amplicon testing performed using male and female fish^9^.

We next sought to determine if the mate-pair architecture of the sequenced libraries could be used to assign males into sex lineages. Existing sex lineage assignments were available for most of the 19 animals, based on microsatellite segregation data obtained from the Tasmanian population. This earlier work revealed *SEX* mapped to either Ssa02, Ssa03 or Ssa06^7^, and the designation used here for each sex lineage is SL-02, SL-03 and SL-06. Eight male fish sequenced in this experiment where assigned to SL-2 (n = 2), SL-03 (n = 2) or SL-06 (n = 4) while the remaining two animals had no assignment (Table 1). Further, microsatellite analysis assigned the Norwegian male used in construction of CHORI-432 to SL-02^9^. Inspection of two TAS SL-02 males revealed the presence of multiple mate pairs spanning the MSR – MFCR junction point at base pair 13,333 (Fig. 4). The spacing of reads and their relative head to tail orientation, when mapped against the SL-02 BAC contig, confirmed the *sd*Y cassette is located on Ssa02 in these animals. Inspection of two SL-03 males and all four SL-06 males revealed a different mate-pair architecture. No instances were observed in any of these individuals of mate pairs spanning the junction point with the correct orientation and spacing. This strongly suggests the *sd*Y cassette in these individuals is not arranged in the contiguous Ssa02 sequence represented by the BAC contig. Rather, orphan reads were identified mapping within the MSR immediately adjacent to the junction point. The mate pair of each orphan mapped elsewhere on the reference genome. Figure 4 illustrates this for animal 2012005062 (bottom panel) which has 6 orphan reads, 4 of which mapped to Ssa03 and the other two map to Ssa06. This suggests the *sd*Y cassette in animal 2012005062 is likely located on one of these two chromosomes, however the sequence data alone was not able to distinguish between Ssa03 and Ssa06. The sex lineage assignment derived from whole genome sequence (SL_WGS) for each animal is recorded in Table 1.

### Male Specific Region SNP

**Figure 5:**
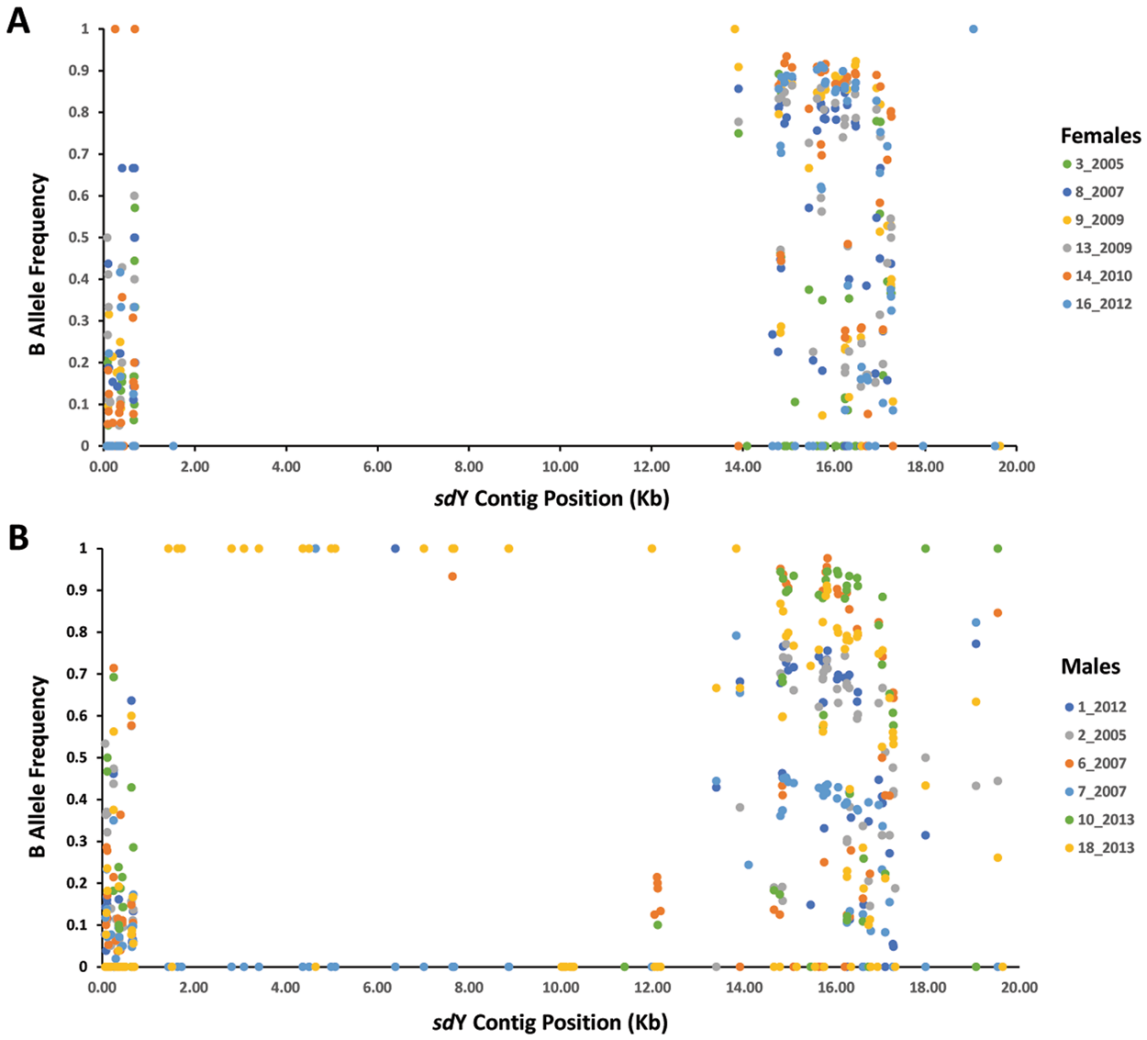
SNP allele frequency spanning the male specific region in male and female fish. B allele frequency (BAF) is shown for 163 SNP distributed across the 20 Kb contig containing the *sd*Y gene. BAF represents the number of reads that carry the B allele as a proportion of the total number of reads observed per SNP within a single animal. Homozygous genotypes therefore take values of 0 or 1. BAF is plotted separately from six female (**A**) and six male fish (**B**).

Variants were called within reads mapped to the 20 Kb *sd*Y BAC contig for each animal. In order to visualise homozygosity and heterozygosity across the region, genotypic status at each locus was assessed as B allele frequency (BAF). This reflects the proportion of reads that carry the alternative (non-reference or B) allele. BAF takes values surrounding 0.5 for heterozygous positions and one or zero for homozygous genotypes. Figure 5 shows the BAF for 163 SNP, plotted separately for males and females. Female fish lacked SNP across the majority of the region (Kb position 2–14) due to the presence of the male specific region (MSR). Inspection of variants within males revealed 99.7% of genotypes in the MSR had BAF values of zero or one, indicating homozygosity for either the reference or alternate allele. One MSR region SNP (Kb position 7.640) in one fish (6_2007) had a BAF slightly less than 1 (0.933) due to the presence of a single read carrying a reference allele. The BAF data (Fig. 5) clearly showed that for the region lacking SNP in females, males were homozygous. Further, the depth of coverage contained in the sequence data spanning the MSR was approximately half the genome wide average in males. Both observations are consistent with sex determination in Atlantic salmon involving male heterogeneity^4^. Specifically, the finding that males lack heterozygosity confirms they carry a single copy of the chromosomal region containing *sd*Y, which is consistent with an XY/XX system for male/female determination.

### Male Specific Region Haplotypes Form Two Haplogroups

**Figure 6:**
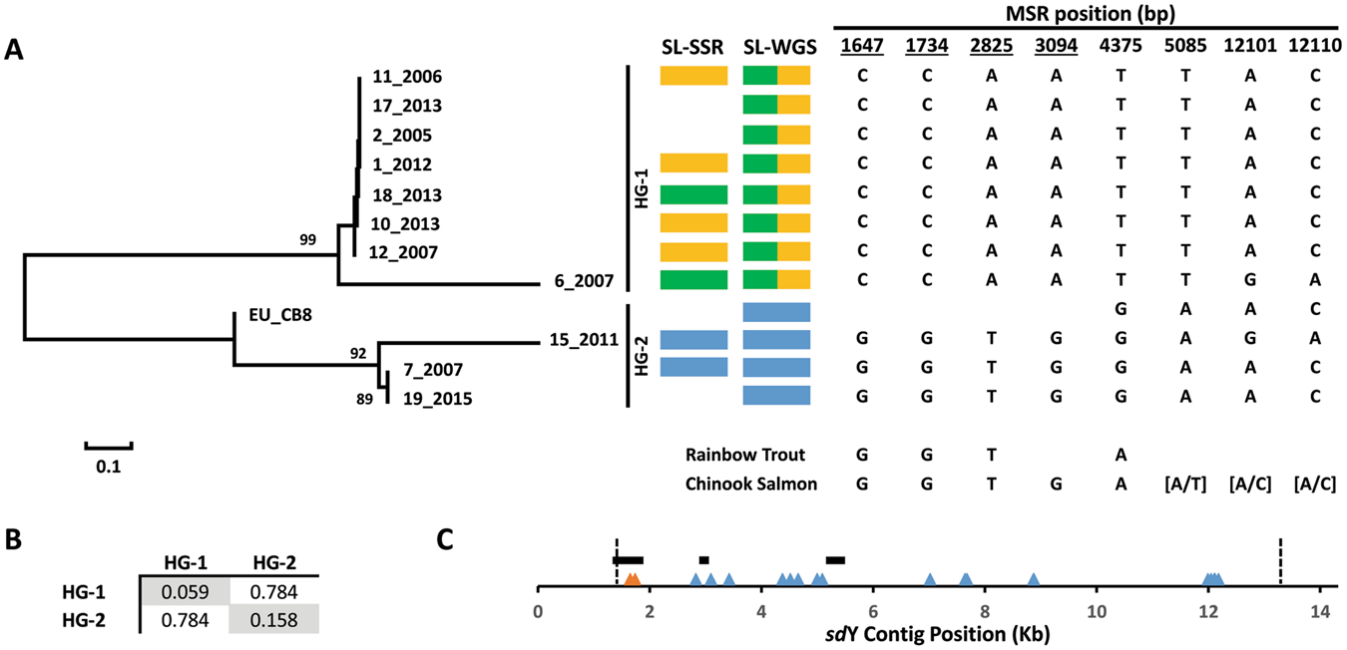
Male specific region SNP haplotypes. Male Specific Region SNP haplotypes were used to estimate evolutionary distance (substitutions per site) and construct a neighbor-joining tree linking male fish (**A**). Animal identifiers are shown to the right of each branch, and nodes with bootstrap replication exceeding 80% are shown to the left. The topography of the tree revealed two clear haplogroups, named HG-1 and HG-2. The assignment of males to sex lineages, using either simple sequence repeats (SL_SSR,^7^) or whole genome sequencing (SL-WGS, this study), is indicated by blue (SL-02), green (SL-03) or yellow (SL-06) blocks. WGS was unable to discriminate SL-03 from SL-06, and is therefore represented by both green and yellow. Alleles are shown for 8 positions where homology testing assigned the nucleotide present in either Rainbow trout or Chinook salmon. Phylogenetically informative positions relating to the ancestral state in Atlantic salmon are underlined, and those retaining polymorphism are denoted using square brackets. (**B**) The genetic distance present within each haplogroup is given as the mean number of substitutions per polymorphic site (grey boxes). The distance separating the haplogroups is given using the same metric. (**C**) A total of 22 polymorphic MSR sites were used to construct the tree. Their position within the 20 Kb *sd*Y contig is shown using triangles. *sdY* exons 2–4 are shown using back triangles. The boundary of the MSR is indicated using vertical dashed lines. Two SNP (orange) overlap with *sd*Y exon 2 while the remaining 20 are intergenic (blue).

The homozygosity of MSR variants in male fish defined a haplotype for each animal. To explore the divergence between haplotypes, pairwise genetic distance was used to construct a Neighbor-Joining tree (Fig. 6A). A clear bifurcation was observed, separating haplotypes into two distinct haplogroups (termed HG-1 and HG-2) with high confidence (99% bootstrap value across 1000 replications). The divergence between haplogroups was high, with a mean value of 0.78 substitutions per polymorphic site separating HG-1 haplotypes from those in HG-2 (Fig. 6B**)**. To visualise the relationship between haplogroups (HG-1, HG-2) and sex lineages (SL-02, SL-03 or SL-06), the microsatellite and WGS derived assignments for each animal are provided graphically in Fig. 6. This revealed a perfect correspondence between haplogroup and sex lineage membership, whereby HG-1 contains SL-03 and SL-06 males while HG-2 contains SL-02 individuals. Two observations are noteworthy. First, this suggests a deep evolutionary split between MSR sequence of SL-02 animals compared with either SL-03 or SL-06 males. Secondly, SL-03 and SL-06 males carry haplotypes with little or no divergence separating them. This suggests a recent event has generated one from the other, (SL-03 from SL-06 or SL-06 from SL-03) however it is not currently clear in which direction this occurred. The mechanism is also not certain, however the presence of intergenic *sd*Y mariner TC1 elements has been used to suggest transposon mediated gene transfer has occurred^19^. Alternatively, recent homeologous recombination would also explain the lack of haplotype divergence, and our mapping results revealed the Ssa03 and Ssa06 loci are located in homeologs with elevated sequence similarity.

Reconstructing the evolutionary history of the three identified sex determination loci requires knowledge concerning which is likely to be ancestral and shared with other salmonid lineages. For each of the 22 MSR variants used to define HG-1 and HG-2, we performed homology analysis in an effort to determine the allele present in both Rainbow trout (*Oncorhynchus mykiss*) and Chinook salmon (*Oncorhynchus tshawytscha*). Sequence conservation was generally low, with only a small region spanning *sd*Y retaining inter-species homology in agreement with previous findings^19^. We successfully assigned alleles for 8 of the 22 Atlantic salmon MSR variants in at least one of the outgroup species. Four of these are phylogenetically informative for the identification of the *Salmo salar* ancestral state (Fig. 6). At all four loci, the allele present in the outgroup species is identical by state with the haplotype carried by SL-02 males that have their MSR located on Ssa02. This is strong preliminary evidence indicating the Ssa02 SD locus is ancestral, and the Ssa03 and Ssa06 loci are derived. Given the very low divergence separating SL-03 and SL-06 haplotypes, it is almost certain the Ssa03 and Ssa06 loci have diverged from each other after the separation of Atlantic salmon from other salmonid lineages. It is not clear if the older event, which defines SL-02 as separate from SL-03 and 06, predates the emergence of Atlantic salmon from other lineages. This leaves open the possibility that members of the *Oncorhynchus* genus may also have multiple SD loci that remain to be identified by WGS and GWAS.

## Discussion

In this study, we used two complimentary approaches to discover and characterise loci responsible for sex determination in Atlantic salmon. GWAS and SNP array data were used to identify three separate SD loci in the TAS breeding population, and locate them to broad chromosomal segments. GWAS identified more than three regions, and evidence was presented to suggest the signals on Ssa05 and Ssa12 are spurious, however it is possible the association detected on Ssa25 may represent an additional SD locus. GWAS was unable to define narrow critical intervals for any of the loci despite the experiment exploiting ample loci and animals (46,501 SNP and >4000 fish). This likely arose due to the non-uniform genomic distribution of male recombination^14^ and linkage disequilibrium that persists over hundreds of Kb^10^. The other approach we adopted provides complimentary data via WGS. Mapped read depth compared between males and females precisely delineates the junction point separating the MSR from the male – female common region. WGS was not, however, able to place the chromosomal location of the MSR onto the female derived reference genome assembly. This is likely to require application of long read WGS technology such as PacBio or Oxford Nanopore using male fish DNA, to traverse the repetitive elements that flank the MSR and read into unique copy regions present in ICSASG_v2^9^. Patterns of WGS derived SNP variability report two important results. Firstly, B allele frequency analysis shows males are homozygous across the chromosomal region absent in females. This confirms males carry a single copy of the MSR containing *sd*Y. Previous evidence in support of male heterogameity is indirect, arising from the observation that mating hormonally sex reversed females with normal females generates all female offspring^20^. Sequencing male fish, and aligning their reads against the *sd*Y containing contig, provides direct experimentation confirmation that Atlantic salmon rely on an XY/XX system. Secondly, WGS facilitated comparison of the MSR haplotypes carried in males from three different sex lineages. This identified the haplotype carried by SL-02 animals is likely the ancestral version through comparison with other salmonids. Interestingly, no clear divergence was observed between the haplotypes carried by SL-03 and SL-06 males, suggesting the translocation event that founded one from the other is likely to be recent. This is unexpected, given all three sex lineages are present in both North American derived (this study) and European stocks which are estimated to have diverged 500–600 thousand years ago^21^. This apparent contradiction could be explained by i) gene flow between the two subpopulations; ii) a mutation rate in the MSR so low that the SL-03 and SL-06 haplotypes are indistinguishable after hundreds of thousands of years or iii) that the genomic location of SD loci is not the same in NA and EU populations. Given the failure in this study to accurately position the SD loci in NA derived populations, coupled with the current paucity of mapping data in EU populations, it remains an open question if the loci are identical by state in the two subpopulations.

In conclusion, we have characterised multiple sex determination loci in Atlantic salmon to further our understanding of a trait which has both ecological interest and important consequences for aquaculture production.

## Methods

### Genome Wide Association Study

The GWAS population is from the SALTAS selective breeding program, described elsewhere^7,10,22^. All animals used in this study were part of the commercial operations of Tassal and Saltas, and their use was in accordance with authorised management practises of both companies and compliant with the Tasmanian Animal Welfare Act (1993). A total of 582 families were generated using 2012, 2013 and 2014 year class broodstock, before the sex of their progeny was assigned using two methods. Phenotypic sex (PSEX) was recorded for 3176 fish by a mix of visual assessment of developing animals in freshwater and dissection at harvest in marine animals. Genotypic sex (GSEX) was assigned in 4715 fish using intensity data from three probe sets designed to detect the presence of exon 3 and exon 4 of *sdY* (Supplementary Table S1**)**. Animals were assigned as male where each *sdY* assay returned a positive signal, and female where no intensity data was observed from any *sdY* assay. DNA samples were extracted from fin clip tissue and genotyped using a custom Tasmanian Salmon 50 K Affymetrix SNP array developed by the Center for Aquaculture Technologies (San Diego, California). The SNP content is largely derived from a custom 220,000 SNP Affymetrix array used previously for genotyping the TAS population to identify segregating and polymorphic loci [10]. Raw genotype calls were assessed by cluster analysis to remove failed loci and those with poor cluster separation. Data was then filtered to remove: SNP with call rate < 90%; SNP with minor allele frequency < 1% and individuals with greater than 5% of missing genotypes. A comprehensive pedigree check was performed by comparing the coefficients of the additive relationship matrix and the genomic relationship matrix (G matrix) calculated via the first method described in^23^. Thirty nine fish were identified with multiple inconsistencies and removed. After all filtering, 46,501 SNP and 4716 animals were used for GWAS. Analysis was carried out using PLINK v1.9^24^. Population substructure for genotyped fish was examined with no significant stratification observed. Binary trait allelic association (case-control) was performed using linear regression before the results were adjusted for multiple testing for each trait (–*logistic*–*adjust*). SNP were mapped to ICSASG_v2 to facilitate plotting of SNP significance in genomic order. PLINK v1.9 was also used to assess non-syntenic linkage disequilibrium for SNP pairs drawn from Ssa02, Ssa05 and Ssa12. All pairwise *r*^2^ values were reported after setting the LD window to 0.0 (*–ld-window-r2*) and LD was reported for inter-chromosomal pairs using*–inter-chr*.

### Samples, Sequencing and Read Mapping

A total of 20 animals were sampled from the SALTAS breeding program to present a range of year classes, families, phenotypic sex and sex lineage as summarized in Table 1. Six individuals had previously been genotyped using a custom 220000 SNP Affymetrix array^10^, and were included to facilitate concordance analysis of variant calling. Samples were stored as fin clips under ethanol before DNA extraction was performed immediately prior to the commencement of genome sequencing. A total of 2.5 ug of genomic DNA per fish was used for construction of short insert libraries in preparation for paired-end sequencing to generate 2 × 150 bp reads on Illumina’s HiSeq X Ten system. To compare the TAS animals with other populations, genome sequence from 20 additional farmed Atlantic salmon were downloaded from the SRA (SRP059652). These have been described previously during construction of an Affymetrix Custom SNP array^12^. Seven are farmed Chilean fish of North American origin (referred to as NA throughout), and include three individuals from the Cascade strain originating from Gaspe Bay, Saint Jean River (Quebec, Canada) and four from Nova Scotia (Canada). It is worthwhile noting the TAS population derives from wild stock sampled from the River Phillip, also located in Nova Scotia. The remaining 13 animals are Chilean of European origin (EU), originating either from Scotland (Lochy and Landcatch strains) or Norway (Fanad and Mowi strains). Data from Illumina whole genome sequencing (WGS) were quality trimmed using quadtrim version 2.0.1 with parameters -q 20 -a 20 -l 75 -p 3 (Trim bases with phred score less than 20 from 3′ and 5′ ends, discard reads with mean phred score less than 20, remove reads that have 3 or more N in the sequence, remove reads less than 75 bp). A 20 Kb contig spanning *sd*Y^9^ was appended to reference ICSASG_v2 as an unmapped scaffold, to facilitate read alignment to the male specific region (MSR) missing from the female derived reference assembly. It is worthwhile noting this approach offered every read the opportunity to map either to the female reference assembly or the 20 Kb BAC contig. If reads were only mapped against the 20 Kb contig, a higher proportion of reads mapped to the male specific region in female fish (data not shown). Trimmed sequences were mapped to the *Salmo salar* reference ICSASG_v2 (NCBI accession GCA_000233375.4) using BWA mem with default parameters. Duplicates sequences were removed from the resulting BAM files using samtools rmdup (version 1.3.1). Local realignment around indels was conducted using GATK IndelRealigner (version 3.6.0).

### Variant Calling and Concordance Analysis

Variants were called using GATK Haplotype caller to produce GVCFs subsequently used for joint genotyping from all samples to produce a merged VCF file for all 40 genomes. Variants were filtered using bcftools filter (version 1.3.1) to remove variants: i) with mapping quality < 50; ii) read depth < 5 and iii) variants other than biallelic SNP. Data from six individuals independently genotyped using a custom 220000 SNP Affymetrix array were extracted for use in concordance analysis to assess the quality of WGS variant calling. To define a collection of variants with both WGS derived and array based SNP calls, loci on the Tasmanian Salmon 50 K Illumina SNP array were first mapped to ICSASG_v2 to obtain base pair coordinates. Comparison against WGS SNP identified 16,436 overlap loci suitable for concordance testing. WGS derived SNP were converted to a numerical format (0,1,2) for comparison to array based data, before each genotypic outcome underwent concordance analysis.

### Population Divergence: PCA, RAF and F_ST_

For comparisons between populations (TAS, EU and NA), down sampling was performed to normalise for read depth. TAS genome data was down sampled using GATK version 3.6.0 Printreads (-dfrac = 0.2) before variant calling was performed for each individual using GATK Haplotype caller. Population diversity was assessed as nucleotide diversity (π) using genome sequence, estimated using Vcftools v.01.14 in 20 kb genomic bins with a 10 kb step window (–*window-pi 20000*–*window-pi-step 10000*). Reference allele frequencies within population were estimated using Vcftools (–*freq*). Population divergence, measured as *F*_ST_, was calculated using–*weir-fst-pop*. Pairwise values were used as the distance metric in neighbour-joining tree construction in R (v 3.2.5) using the library ape. PCA was performed in PLINK v1.9 following LD based SNP pruning with *r*^2^ threshold of 0.3 (*–indep-pairwise 500 5 0.3*).

### MSR BAF and Haplotype analysis

Raw variants called using the 20 Kb *sdY* BAC contig were filtered to remove indels and SNP with read depth of 5 or lower. B allele frequency (BAF) was estimated directly from the resulting VCF by dividing the alternate allele count by the read depth for each SNP in each animal. Filtered SNP were considered to be located in the MSR because they were i) missing in every female and ii) present in 95% of the TAS males sequenced. Haplotypes were used for the construction of a Neighbor-Joining tree using MEGA5^25^. Positions were used for estimation of a distance matrix following removal of positions call rate <75% per SNP and missingness across the collection of male fish >50%. This identified 22 variants in 12 MSR haplotypes used for evolutionary distance estimation using the p-distance method with units of base differences per polymorphic site. Neighbor-Joining tree robustness was assessed using the bootstrap test (1000 replicates).

### Data Availability

Genome sequence of 19 Tasmanian salmon has been deposited to NCBI as BioProject ID PRJNA403334 and individual animal raw sequence datasets are accessioned as SRR6019467 - SRR6019464. Variants called from the 19 animals are available in Dryad Digital Repository with DOI 10.5061/dryad.117hh. The BAC contig used for interpretation of the male specific region is available as Genebank accession KP898412. Genome sequence for the double haploid fish used for variant filtering are accessioned as PRJEB24419 at the European Nucleotide Archive. To compare the TAS animals with other populations, genome sequence from 20 additional farmed Atlantic salmon were downloaded from the SRA (SRP059652). Trimmed sequences were mapped to the Salmo salar reference ICSASG_v2 (NCBI accession GCA_000233375.4). All remaining data is available upon reasonable request for the authors.

## Electronic supplementary material

Supplementary Information
