# Supplementary material for: Evolution of Sex Determination Loci in Atlantic Salmon
Source: Sci Rep. 2018 Apr 4;8:5664. doi: 10.1038/s41598-018-23984-1 (PMC5884791; doi:10.1038/s41598-018-23984-1)
Supplement: Supplementary file 1 — Supplementary Information [file 41598_2018_23984_MOESM1_ESM.pdf]

Supplementary Information

Evolution of Sex Determination Loci in Atlantic Salmon

James Kijas[1*], Sean McWilliam[1], Marina Naval Sanchez[1], Peter Kube[2], Harry King[2], Bradley Evans[3], Torfinn Nome[4], Sigbjørn Lien[4] and Klara Verbyla[5]

Affiliations:

[1] CSIRO Agriculture & Food, St. Lucia, 4067, QLD, Australia. [2] CSIRO Agriculture & Food, Hobart, 7004, TAS, Australia. [3] Tassal Operations, Hobart, 7000, TAS, Australia. [4] Centre for Integrative Genetics (CIGENE), Department of Animal and Aquacultural Sciences, Faculty of Biosciences, Norwegian University of Life Sciences, P.O. Box 5003, N-1432 Ås, Norway. [5] CSIRO Data 6, Canberra, 2601, ACT, Australia. * Corresponding Author: James Kijas

James.Kijas@csiro.au

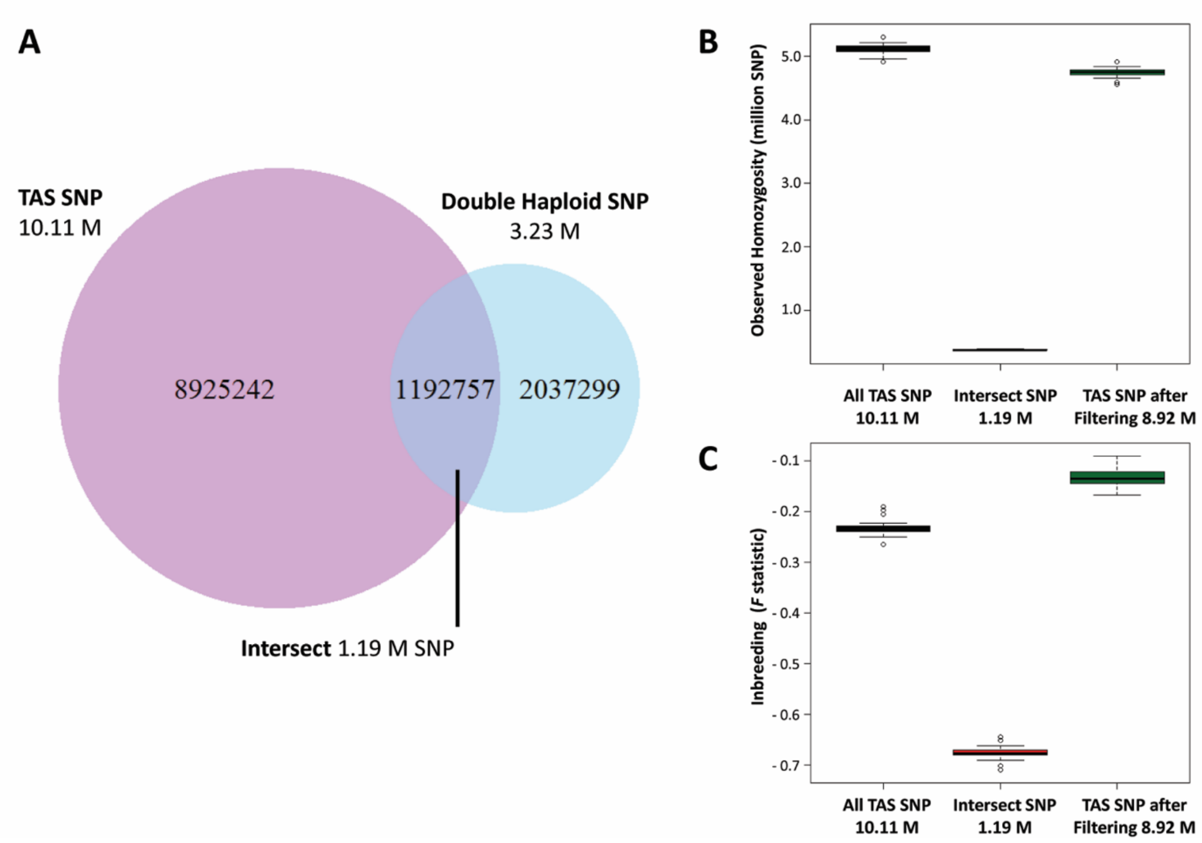

Supplementary Figure S1. Quality filtering using double haploid genome sequences. A collection of 10.11 M SNP, called within 19 Atlantic salmon from Tasmania (TAS), were evaluated for the presence of loci independently identified by genome sequencing four double-haploid fish (a). These four haploid animals were generated by mitotic androgenesis, as described in an earlier study [11] and given their haploid status contain no SNP. They were used to identify 3.23 M positions that falsely appear as variable loci in any of the four individuals, likely arising as a consequence of the whole-genome duplication events that characterize salmonids [11]. Comparison between the two SNP collections (TAS and double-haploid) identified 1.19 M positions in common (or 12% of TAS SNP). These were subsequently filtered out of the TAS collection, leaving a total of 8.92M that were used in all subsequently analysis. Two population metrics were estimated and compared when using all TAS SNP (10.11 M), the intersect subset common to the double haploid SNP (1.19 M) and the

TAS collection after filtering (8.92 M). Observed homozygosity (b) revealed the intersect SNP contained very few homozygous loci. These positions were nearly all heterozygous, which is consistent with fixed differences within duplicated genome regions presenting as heterozygous during variant calling. The inbreeding estimate (c) increased following the removal of the intersect SNP, reflecting the extraction of a large number of heterozygous datapoints.

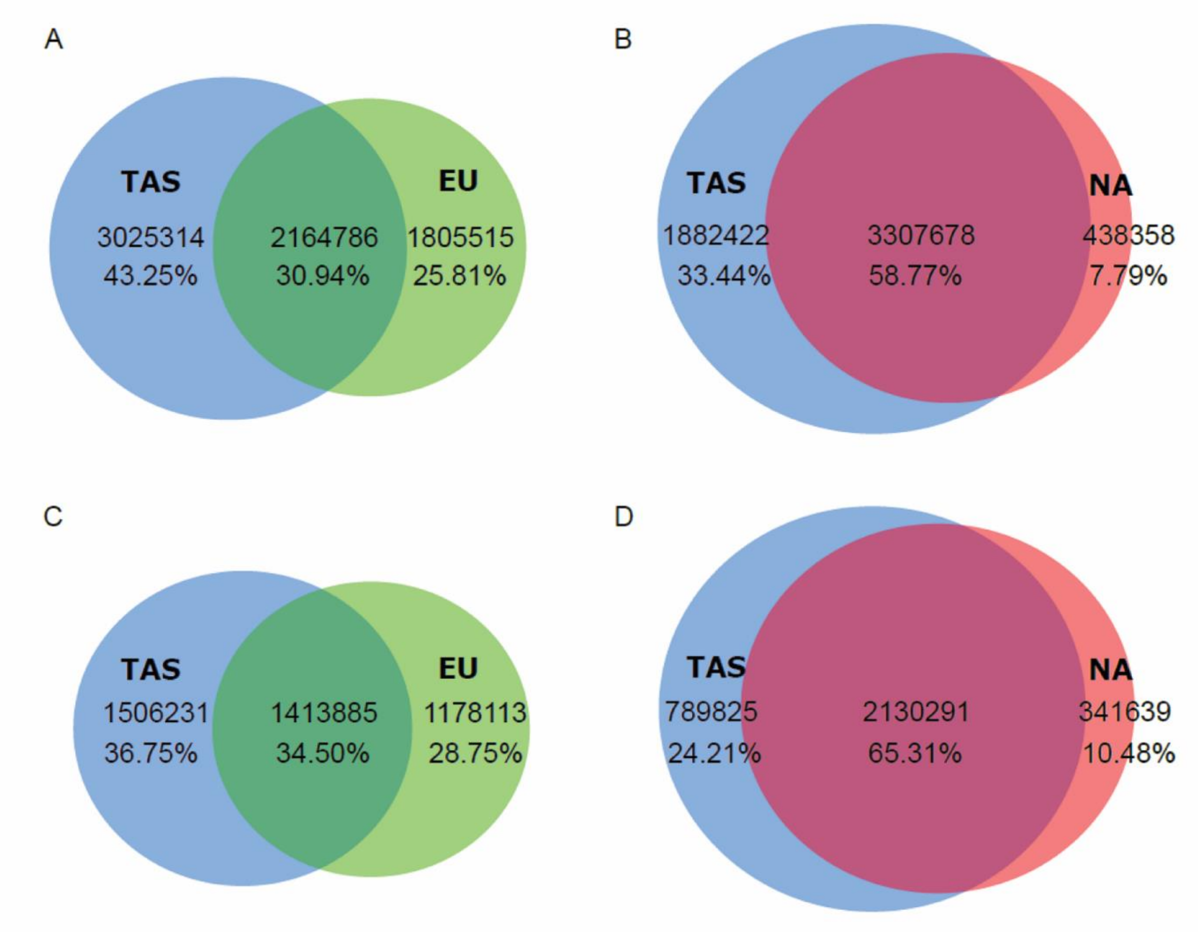

Supplementary Figure S2. Shared SNP between populations. Shared and private SNP comparing Tasmanian (TAS), European (EU) and North American (NA) derived captive Atlantic salmon. Following variant calling within population, SNP were defined as being either i) private to a particular population or ii) shared between populations. Shared variants were identified at the same nucleotide position, with the same alternate (non-reference) allele in multiple populations. The TAS animals were compared against the EU (a and c) and NA animals (b and d) separately. Comparison between the TAS and NA populations revealed nearly half of the variants were private to the Tasmanian fish (44%) while only 25% were found private within the European animals (b). Given the depth of coverage available for variant discovery in the TAS genomes (33 – 48 x) is much higher than the NA and EU data (7 – 10 x), corrective read subsampling was performed on the TAS data before variant calling was repeated using matched lower depth of coverage (c and d). This reduced the total number of

variants identified by 15%, and the proportion of private alleles within the Tasmanian animals to 37% of the total (c). Given the effect of the correction for read depth was meaningfully large, it was applied to each estimate reported in this study that directly compares populations.

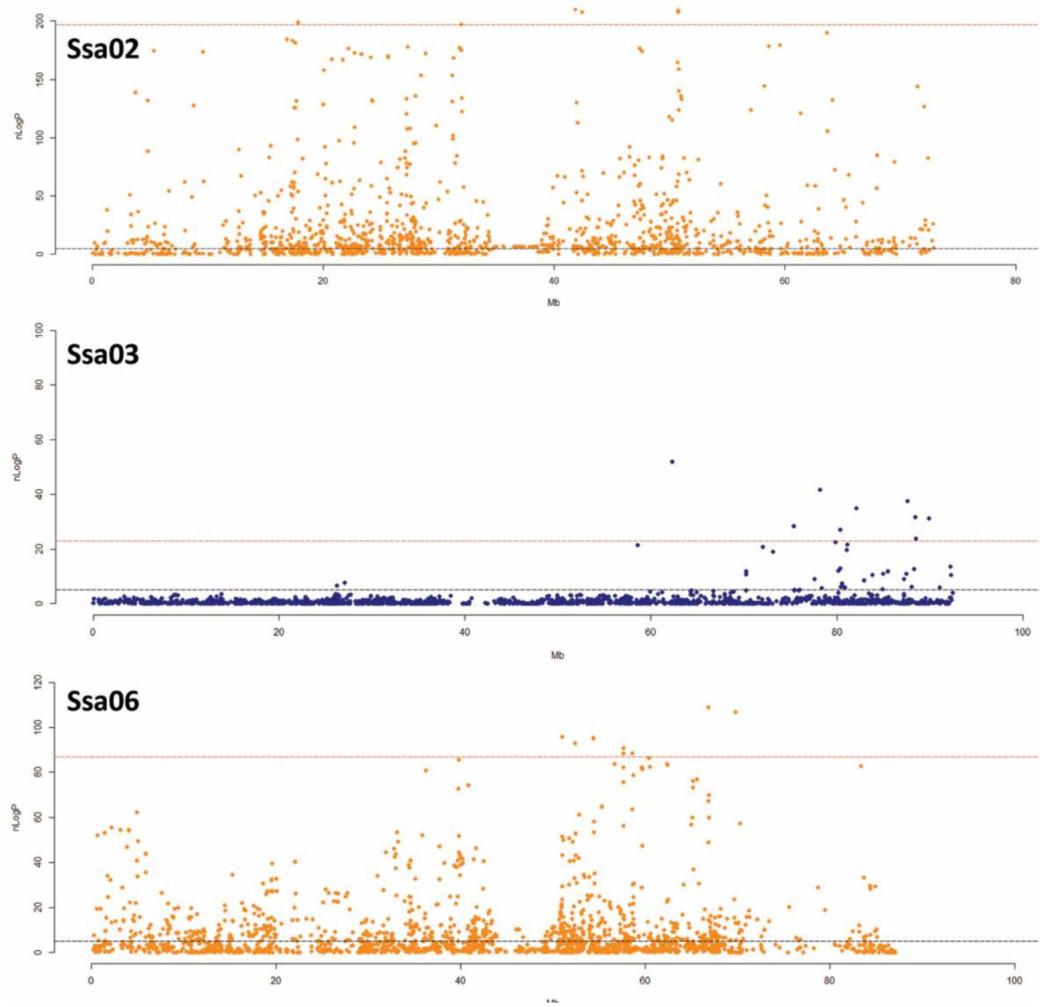

Supplementary Figure S3. GWAS for three Atlantic salmon chromosomes. A total of 46,502 SNP were used for association analysis to identify sex determination loci in a population of 4715 fish scored for genotypic sex (GSEX). Strong association signals were identified on chromosomes 2, 3 and 6, shown here with chromosomal specific thresholds (red lines). SNP associations for all chromosomes are shown in Fig 2.

**Probe Set**	**Target Sequence**
Exon4_sdy	CCATGGGCTCAGCAGCTATTCAAGCAAGCTCACGA[C/T]TTCAGGATCTGGCTTGAGTCCTCCCCTGTCTCTCC
Exon3_sdy	AGCAGAGCAGATGGCTTCCAACCGCAAATTGGGTT[C/T]AGCCTATGGTTCGGACAAGACTCATCACTCAGTGC
SJ1	TGATGCTCTTCAACAGGGAGACCTTCAGACAGGGT[G/T]AGTGATATTAAGGGAATCTGTTACGGGTAATTCAC

Supplementary Table S1. GSEX *sdY* Probes. Three regions were targeted using probes incorporated into the Tasmanian Salmon SNP50 genotyping platform. This facilitated assignment of GSEX for animals genotyped using the SNP array
